# Molecular Characterization of the Monoclonal Antibodies Composing ZMAb: A Protective Cocktail Against Ebola Virus

**DOI:** 10.1038/srep06881

**Published:** 2014-11-06

**Authors:** Jonathan Audet, Gary Wong, Han Wang, Guangwen Lu, George F. Gao, Gary Kobinger, Xiangguo Qiu

**Affiliations:** 1 Department of Medical Microbiology, Faculty of Medicine, University of Manitoba, Winnipeg, Canada, R3E 0J9; 2 Special Pathogens Program, Canadian Science Center for Human and Animal Health, Public Health Agency of Canada, Winnipeg, Canada, R3E 3R2; 3 CAS Key Laboratory of Pathogenic Microbiology and Immunology, Institute of Microbiology, Chinese Academy of Sciences, Beijing 100101, P.R. China; 4 Department of Immunology, Faculty of Medicine, University of Manitoba, Winnipeg, Canada, R3E 0J9; 5 Department of Pathology and Laboratory Medicine, University of Pennsylvania School of Medicine, Philadelphia, PA, USA

## Abstract

Ebola virus (EBOV) causes severe viral hemorrhagic fever in humans and non-human primates, with a case fatality rate of up to 88% in human outbreaks. Over the past 3 years, monoclonal antibody (mAb) cocktails have demonstrated high efficacy as treatments against EBOV infection. One such cocktail is ZMAb, which consists of three mouse antibodies, 1H3, 2G4, and 4G7. Here, we present the epitope binding properties of mAbs 1H3, 2G4, and 4G7. We showed that these antibodies have different variable region sequences, suggesting that the individual mAbs are not clonally related. All three antibodies were found to neutralize EBOV variant Mayinga. Additionally, 2G4 and 4G7 were shown to cross-inhibit each other *in vitro* and select for an escape mutation at the same position on the EBOV glycoprotein (GP), at amino acid 508. 1H3 selects an escape mutant at amino acid 273 on EBOV GP. Surface plasmon resonance studies showed that all three antibodies have dissociation constants on the order of 10^−7^. In combination with previous studies evaluating the binding sites of other protective antibodies, our results suggest that antibodies targeting the GP_1_-GP_2_ interface and the glycan cap are often selected as efficacious antibodies for post-exposure interventions against EBOV.

Ebola virus (EBOV) causes severe hemorrhagic fever in humans and non-human primates (NHPs). In past outbreaks, the case fatality rate reached as high as 88%. EBOV is part of the family *Filoviridae*, and forms filamentous viral particles. The only protein present on the surface of the virus is the glycoprotein (GP). The *GP* gene contains a polyadenosine transcription slippage site (genome position 9618–9624, GP position 880–886, amino acids 294–296) and encodes three versions of the EBOV viral glycoprotein. The default protein, made from an unmodified slippage site with 7 adenosine residues (A) is the soluble glycoprotein (sGP). During the transcription process, the viral polymerase may insert extra A residues into this slippage site^1^. The insertion of 2 A or the removal of 1 A, for a total of 9 or 6 residues, leads to the production of the small soluble glycoprotein (ssGP). The insertion of a single A, for a total of 8 residues, results in a frameshift mutation and leads to the production of the full-length trimeric glycoprotein (GP_1,2_; or virion spike protein) with each monomer composed of two subunits, GP_1_ and GP_2_. The GP_1_ subunit (amino acids 33–501) contains the core of the glycoprotein, its receptor binding domain (RBD), a glycan cap, and a large mucin-like domain which extends around the RBD in the form of a chalice^2^. The GP_2_ (amino acids 502–676) subunit contains the internal fusion loop, heptad repeats 1 and 2, the membrane-proximal external region, the transmembrane region, and the cytoplasmic tail^2^. The GP_1_ subunit is responsible for receptor binding and immune evasion, most of it is cleaved by endosomal proteases^3^ to allow the unfolding of GP_2_ and the insertion of the internal fusion loop into the endosomal membrane^4^.

Currently, there are no licensed vaccines or treatments against EBOV. In recent years, we and others have shown that the administration of polyclonal antibodies or combinations of monoclonal antibodies (mAbs) prevent fatal disease when administered to EBOV-infected NHPs^5^,^6^,^7^,^8^,^9^,^10^. Treatment with these antibody-based therapies results in complete survival when administered at 24 hours post-infection. These treatments also provide partial protection when treatment starts as late as 5 days post-infection^6^. More recently, a combination of the best two cocktails (ZMAb and MB-003) called ZMapp™ fully protects animals when the treatment is initiated at 5 days post-infection^11^. Here, we study the binding characteristics of one of those cocktails, ZMAb, which combines three mouse-derived mAbs: 1H3, 2G4, and 4G7^8^. These monoclonal antibodies, raised in mice immunized with a VSV-based EBOV vaccine (VSVΔG-EBOVGP), recognize the GP_1,2_^12^.

We previously performed a basic characterization of the epitopes bound by mAbs 1H3, 2G4, and 4G7 using ELISA and western blots^12^. The data showed that 1H3 recognized sGP and GP_1_ in ELISA, but did not bind in western blots. This suggests that the 1H3 binding site is conformational and in the first 295 amino acids, a region shared by EBOV sGP and GP. The antibody 2G4 did not bind to sGP or GP_1_ alone, recognizing only GP_1,2_ in ELISA; it did not react in western blots. This suggests that GP_2_ forms most of the epitope and that it may be conformational. The antibody 4G7 did not bind sGP, but could bind GP_1_ alone as well as GP_1,2_. It also reacted poorly in western blots, suggesting its epitope is also conformational.

In the current study, we aim to explore the molecular properties of mAbs 1H3, 2G4 and 4G7 in more detail. We analyzed the sequence of the antibody variable regions, and tested the mAbs' potential for cross-inhibition and virus neutralization. Additionally, we present data on the affinity of each antibody for the EBOV GP. Overall, the data presented here, along with that published on other mAb cocktails suggests that protective antibody combinations target both the glycan cap/sGP and the GP_1_/GP_2_ interface.

## Results

### Sequence of the antibodies

**Figure 1:**
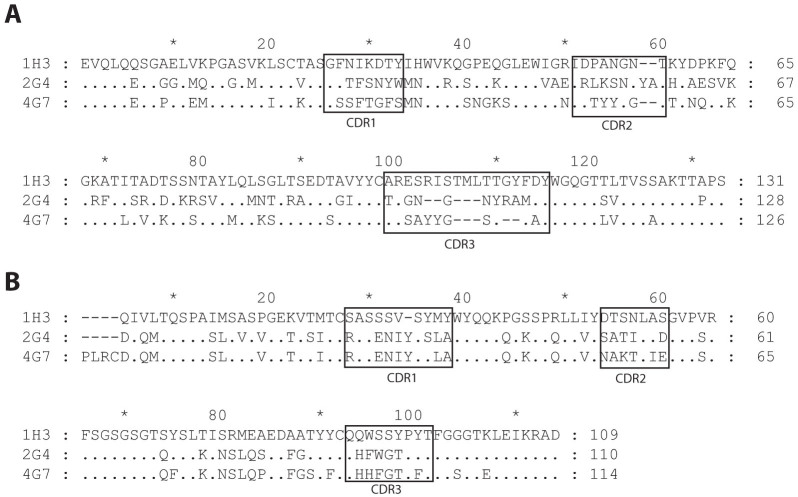
Alignment of the amino acid sequence of the variable regions of the three monoclonal antibodies. The antibodies were sequenced using a nested PCR protocol. The amino acid sequences were analyzed using DomainGapAlign to identify the position of the complementarity determining regions (CDRs). (A) Amino acid sequences of the heavy chain variable regions. (B) Amino acid sequences of the light chain variable regions.

ZMAb consists of three murine antibodies: 1H3, 2G4, and 4G7. The mAbs 1H3 and 4G7 are of the IgG2a isotype, mAb 2G4 belongs to the IgG2b isotype^12^. All three mAbs have a kappa light chain. All three heavy chains are based on different V, D, J germline genes, and show low sequence homology, suggesting the three mAbs are not clonally related (Figure 1A and Table 1). Despite the divergence of the heavy chain variable regions, the light chain variable regions of 2G4 and 4G7 are related to the same family of germline genes and are not very different from each other (Figure 1b and Table 1). The IgBLAST^13^ tool was used to determine the closest germline genes from which each variable region is derived, it presents the top three closest results for each variable region. Given how similar the sequences for the 2G4 and 4G7 light chains are it is interesting to note that the top three closest germline genes for 2G4 were reported as 12-41, 12-44, 12-42 from closest to farthest and that the IgBLAST for the 4G7 light chain gives the three closest germline genes as 12-42, 12-44, 12-41. At the amino acid level, 2G4 and 4G7 share 84.5% identity (Figure 1b).

### Affinity measurement

**Figure 2:**
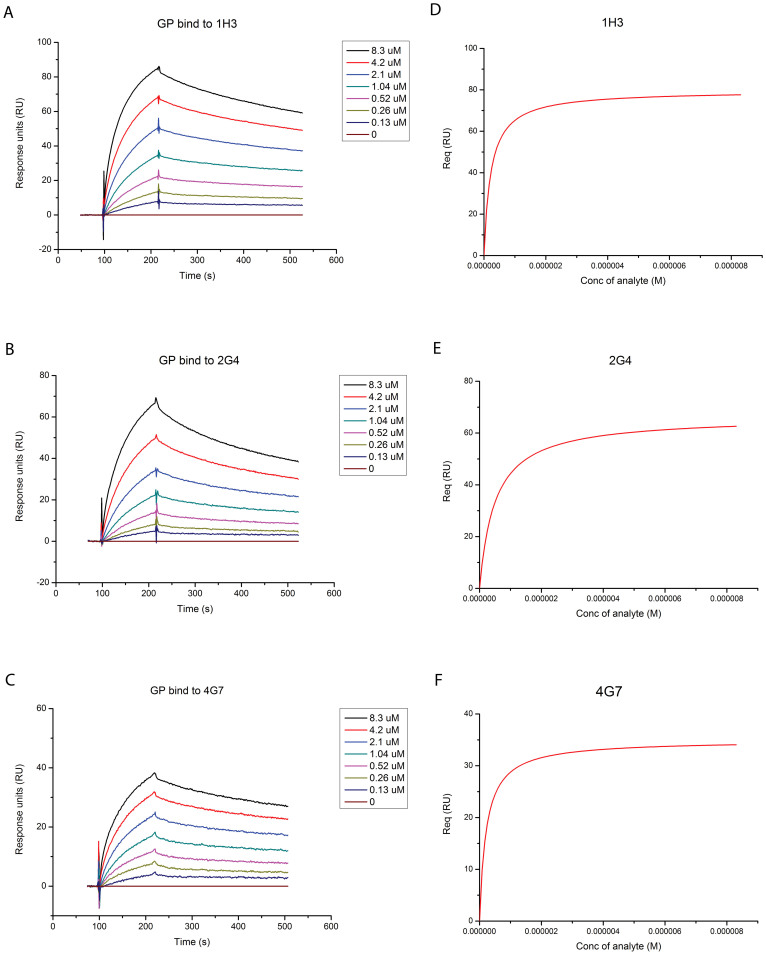
Measurement of the affinity of each mAb for the EBOV glycoprotein (GP). The antibodies were fixed on the BIAcore chip and the specified concentrations of EBOV GPΔTM were run. Measurements for (A) 1H3, (B) 2G4, and (C) 4G7. Fitted Langmuir model for (D) 1H3, (E) 2G4, and (F) 4G7.

To determine the affinity of mAbs 1H3, 2G4 and 4G7 to EBOV GP, the antibody was immobilized and put in the presence of varying concentrations of EBOV GP (Figure 2a–c). The response was fit with a Langmuir binding model (Figure 2d–f). The dissociation constant for 1H3 is 2.11 × 10^−7^ M (χ^2^ = 3.87; df = 2; p = 0.144). The constant for 2G4 is 4.27 × 10^−7^ M (χ^2^ = 2.54; df = 2; p = 0.281). The constant for 4G7 is 2.15 × 10^−7^ M (χ^2^ = 1.14; df = 2; p = 0.566).

### Cross-inhibition

To understand the spatial relationship between the three mAbs, their capacity to block the binding of the others was evaluated in an ELISA-based cross-inhibition assay. 1H3 did not interfere with the binding of 2G4 and 4G7, removing no or 3% of the signal (Table 2). 2G4 and 4G7 also had minimal effect on the signal from 1H3, reducing it to 96 and 91%, respectively. The signal from 4G7 was reduced to 66% by the presence of 2G4 and the signal from 2G4 was reduced to 44% by the presence of 4G7.

### Neutralization

**Figure 3:**
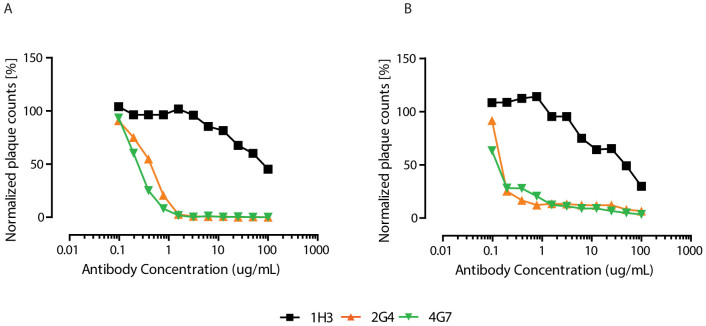
Neutralizing activity of the three antibodies. (A) Neutralization of of VSVΔG-EBOVGP-eGFP by fluorescent plaque counting. (B) Neutralization of EBOV/May-eGFP by fluorescent plaque counting. The results are expressed as normalized plaque count, where 100% was set to incubation of cells with virus only, i.e. in absence of mAbs.

The neutralization potential of the individual mAbs was first evaluated in two systems, first with a VSV virus pseudotyped with EBOV GP and expressing eGFP (VSVΔG-EBOVGP-eGFP) and with a laboratory-developed variant of EBOV expressing eGFP (EBOV/May-eGFP). eGFP-expressing viruses were used in order to compare the neutralization and because plaques formed by EBOV are normally very difficult to visualize and count. The antibodies 2G4 and 4G7 neutralize VSVΔG-EBOVGP-eGFP down to less than 1% of its normal plaque count starting at 3 μg/ml, whereas 1H3 only reduces the plaque counts to 45% even at a concentration of 100 μg/ml (Figure 3A). For EBOV/May-eGFP, 2G4 and 4G7 reduce the plaque counts to 13 and 11% at 3 μg/ml and to 6 and 3% at 100 μg/ml, and again 1H3 only reduces the plaque counts to 29% at 100 μg/ml (Figure 3B). The EBOV/May-eGFP neutralization curves were fitted to a one-phase decay to estimate the antibody concentration needed for 50% neutralization, this concentration was estimated to 27.6 μg/ml for 1H3 (95% CI 15.7-Infinity), 0.139 μg/ml for 2G4 (95% CI 0.129–0.152) and 0.135 μg/ml for 4G7 (95% CI 0–0.199).

### Escape mutation

**Figure 4:**
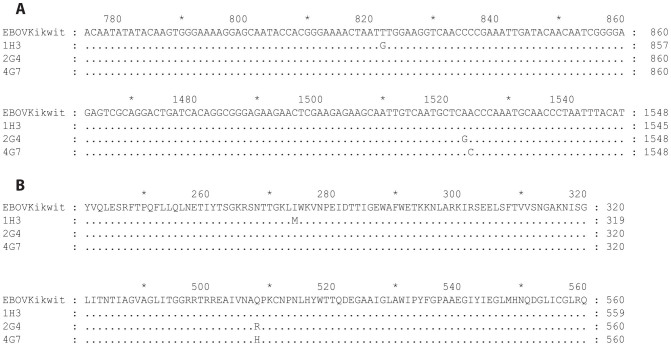
Escape mutants to the monoclonal antibodies 1H3, 2G4, and 4G7. VSVΔG-EBOVGP was passaged three times in the presence of either of the monoclonal antibodies. The EBOV GP gene was then sequenced by Sanger sequencing and aligned to that of the original virus (sequenced in parallel). (A) Alignment of the nucleotide sequences showing the three escape mutations at positions 822 (1H3), 1523 (2G4), and 1524 (4G7). (B) Alignment of the amino acid sequences showing the three escape mutations at positions 274 (1H3), and 508 (2G4 and 4G7).

**Figure 5:**
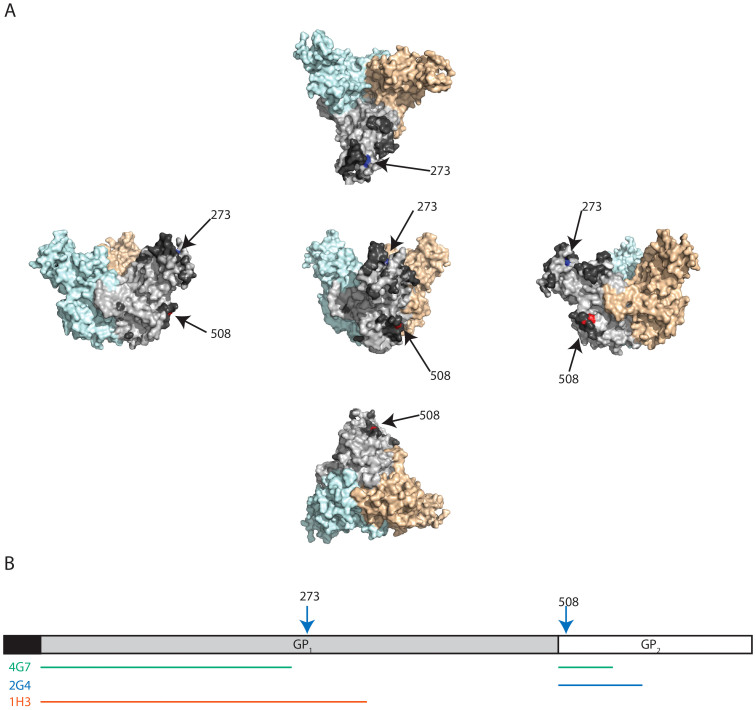
Binding sites of the monoclonal antibodies 1H3, 2G4, and 4G7. (A) Representation of the position of the escape mutations on the EBOV GP structure (based on PDB# 3CSY) from 5 different angles. The escape mutation to 1H3 (aa 273) is identified in blue. The escape mutation site for 2G4 and 4G7 (aa 508) is identified in red. The monomer where the two amino acids are identified is light gray and the other two monomers are light blue and light orange. The dark gray amino acids (only identified on the light gray monomer) were the amino acids with the top 20% antigenicity score calculated by the Epitopia server. (B) Representation of the position of escape mutations (arrows) and suspected binding sites (lines) on a linear map of GP. The putative binding sites are based on escape mutation data and data from previous publications.

The possibility of the virus escaping antibody neutralization was evaluated by repeatedly passaging a VSVΔG-EBOVGP in the presence of the antibodies. The pseudotyped VSV platform was used because it allowed us to perform the experiment in a containment level 2 setting. The GP gene was sequenced to determine the mutation sites of escape mutants (Figure 4a and 4b). Viral selection under partial neutralization by 1H3 produced a mutation at amino acid 273 (I to M) of EBOV GP, which is located in the glycan cap of the glycoprotein (blue amino acid, dashed arrow in Figure 5a). This is consistent with previously published data with 1H3 already known to bind sGP, which only shares the first 295 amino acids with GP_1,2_. Selection under neutralization by 2G4 or 4G7 produced mutations at nucleotide positions 1523 and 1524, respectively. Both of these mutations induce a change in amino acid 508 from Q to R (for 2G4) and from Q to H (for 4G7) (red amino acid, solid arrow in Figure 5a).

## Discussion

In this study, we set out to investigate the molecular characteristics of the three anti-EBOV GP monoclonal antibodies, which are part of the protective cocktail ZMAb. We first sequenced their variable regions to ensure that they were indeed of different clonal origin, and studied the relationships between them. We showed that all the heavy chain variable regions are different and originated from different families of germline genes. Our results also showed that 2G4 and 4G7 share much homology in the sequence of their light chain's variable region. The light chains of these two mAbs are so closely related that they share the same top 3 closest germline genes.

Besides their sequence homology, 2G4 and 4G7 appear to compete for close or overlapping epitopes, as revealed by their high levels of cross-inhibition and the fact the glutamine at position 508 is mutated in the escape mutant to both antibodies. While this mutation was characterized in a VSV-based system, the only documented escape mutation to ZMAb in an NHP was a mutation at position 508^14^. The cross-inhibition of 2G4 by 4G7 is stronger than that of 2G4 by 2G4. This may be due to the slightly higher affinity of 4G7 for GP, allowing it to bind the protein more tightly. This difference in affinity could also explain why 2G4 is not as strongly inhibiting of 4G7 binding as 4G7 is of 2G4 binding. Amino acid 508 is also involved in binding with KZ52 which, like 2G4 and 4G7, is neutralizing^2^.

mAb 1H3 is the only one in ZMAb able to bind sGP. Its ability to block the binding of 2G4 and 4G7 is very low to nonexistent. This is supported by the location of its escape mutant, amino acid 273 is located in the glycan cap of the GP, whereas amino acid 508 (which is mutated in the escape mutants of 2G4 and 4G7) is located beneath the mucin domain. In this study, the low neutralization by 1H3 is consistent with previously proposed neutralization mechanisms^15^. In this model, virus neutralization is achieved when an antibody stabilizes the GP_1_-GP_2_ structure and prevents release of the GP_1_ portion and subsequent unfolding of GP_2_ into the endosomal membrane. The cleavage of GP_1,2_ by endosomal proteases removes the mucin domain and part of the glycan cap, including the likely binding site of 1H3. This is confirmed by the fact that 1H3 selected for an escape mutation (at amino acid 273). However, 1H3 has shown good efficacy *in vivo*^9^,^14^,^16^,^17^, which could be due to its ability to bind sGP, or an ability to recruit other immune mechanisms not tested yet, such as antibody-dependent cellular cytotoxicity (ADCC) or complement-dependent cytotoxicity (CDC).

The mutation sites identified for all three antibodies in ZMAb and the associated putative binding sites are consistent with conformational epitope prediction based on the crystal structure using Epitopia. The conformational epitopes identified by the prediction algorithm, which include the mutation sites, are identified in Figure 5a (dark gray amino acids and the 1H3 and 2G4/4G7 escape mutation sites). The data presented here and the results from Epitopia are also consistent with the previously published ELISA data. According to that study, 1H3 can bind both GP and sGP, suggesting that its binding site is located in the first 295 amino acids of EBOV GP (Figure 5b). For 2G4, ELISA results showed it did not react with GP_1_ alone, suggesting that most of its binding site consists of GP_2_ (Figure 5b). For 4G7, the previously published ELISA data showed it could bind GP_1,2_ and GP_1_ alone, which suggests its binding site is composed of both GP_1_ and GP_2_ (Figure 5b).

Two other sets of monoclonal antibodies have been studied for post-exposure protection of NHPs, the cocktail MB-003^6^,^7^ and a combination of the chimeric mAbs ch133 and ch226^18^. The MB-003 cocktail is made of the human-mouse chimeric antibodies 13C6, 13F6, and 6D8. The binding regions of these antibodies had been determined previously^19^. The antibody 13C6 was found to bind both GP_1_ and sGP, a binding profile shared with our antibody 1H3. The antibodies 13F6 and 6D8 were found to bind to linear epitopes in the mucin-like domain of GP_1,2_ (amino acid 401–417 for 13F6, and 389–405 for 6D8). None of the antibodies in the ZMAb cocktail recognize those epitopes. The original mouse antibody 133 was found to induce the production of escape mutants with a mutation at amino acid 549 (in GP_2_), and mAb 226 induced escape mutants at three different positions in GP_1_ (amino acids 134, 194, and 199)^20^. Only one of the escape mutations for mAb 226 can be found on the crystal structure of GP_1,2_, amino acid 134 is located to the side of the glycan cap. The published *in vivo* efficacy of the ch133/ch226 combination was lower than that of MB-003 and ZMAb but the dose and dosing regimen used were also very different. The escape mutation data presented in this study have increased the precision of the binding locations described previously for the three antibodies in the ZMAb treatment. With this additional information, it is interesting to note that all three cocktails include one antibody that binds to or close to the glycan cap (ch226, c13C6, and 1H3) and that two of the three cocktails (ch133/ch226 and ZMAb) include at least one antibody that binds the GP_1_/GP_2_ interface. The fact that three independent groups converged on similar solutions suggests that these two regions may be especially important. This knowledge could be used to choose the antibodies to include in a mAb cocktail directed against related viruses such as Marburg virus or Sudan virus.

The possibility of escape mutations to 2G4 and 4G7, which would allow for unrestricted viral replication, may raise concerns regarding the efficacy of the ZMAb treatment. However, in over 36 non-human primates treated across several studies (24 from published studies, 12 not yet published) only one case of an escape mutant was found, suggesting an occurrence during treatment of 3% or less and no wild-type sequence available from GenBank possesses these mutations. This information suggests there may be a steep fitness cost to these mutations. It is also possible that the presence of two antibodies that bind to overlapping epitopes reduces the odds of an escape mutant.

Overall, the ZMAb antibody combination therapy is composed of three different antibodies. These antibodies bind three epitopes, two of which are probably overlapping as evidenced by their cross-inhibition and the location of their escape mutations. The antibodies with overlapping epitopes, 2G4 and 4G7, are also strongly EBOV-neutralizing. Finally, the three antibodies have similar levels of affinity for the GP, although 1H3 shows the highest affinity, followed closely by 4G7 with 2G4 at half the affinity of the first two. The information presented in this study along with that from previous work by us and other groups suggests a rational decision on the choice of antibodies based on their target is possible.

## Methods

### Viruses

Recombinant Vesicular stomatitis virus (VSV) expressing EBOV GP and eGFP (VSVΔG-EBOVGP-eGFP) was generated as described previously^21^, the eGFP gene was inserted in the MluI and AvrII restriction sites. The eGFP-expressing EBOV used for the neutralizing antibody assay is Ebola virus NML/H.sapiens-lab/COD/1976/Mayinga-eGFP-p3 (EBOV/May-eGFP) (derived from an Ebola virus, family *Filoviridae*, genus *Ebolavirus*, species *Zaire ebolavirus*, GenBank accession No NC_002549), which encodes the eGFP reporter gene between the NP and VP35 ORFs.

### mAbs

The monoclonal antibodies were isolated and produced as previously described^11^,^17^.

### Sequencing

Sequencing was performed using the primers designed by Tiller *et al*.^22^ RNA was extracted from approximately 10^7^ viable hybridoma cells using a Qiagen RNeasy Mini Kit according to manufacturer instructions. Reverse transcription was carried out using the Improm II kit (Promega) with random hexamers as primers. Semi-nested PCR was then performed to amplify the desired regions on mAbs 1H3, 2G4 and 4G7. The first round of PCR was carried out with the heavy chain primers MsVHE, CμOut, Cγ1Out, Cγ2cOut, Cγ2bOut, Cγ3Out, CαOut and the light chain primers mCk, LVk3, LVk4, LVk5, LVk6, LVk689, LVk14, LVk19, and LVk20. The PCR was performed using Phusion Hi-Fidelity PCR kit (Thermo) in a total volume of 50 μl under the following conditions: 98°C for 3 min, 30 cycles of denaturation at 98°C for 1 min, annealing at 50°C for 1 min, and extension at 72°C for 1 min, and then a final extension 72°C for 10 min. The second round of PCR was carried out on 2 μl of the first round reaction. The primers used for the second round light chain amplification were mV, mJK01, mJK02, mJK03, mJK04 and the heavy chain primers were MsVHE, CμIn, Cγ1In, Cγ2cIn, Cγ2bIn, Cγ3In, and CαIn. The PCR conditions were similar to the first round, changing only the annealing temperatures to 45°C the light chain and 60°C for the heavy chain. The resulting products were gel-purified and extracted using a Qiagen Gel Extraction kit according to manufacturer instructions. The purified products were cloned in the pJet vector using the CloneJet kit (Thermo) and sequenced by an in-house sequencing service using the provided vector primers. The sequences were assembled using DNAStar Lasergene Seqman v9. The position of the complementarity determining regions was determined using the IMGT DomainGapAlign tool^23^. The germline genes were determined by using the IgBLAST tool^13^.

### Epitope prediction

Conformational epitopes were predicted using the Epitopia server^24^. This server uses an algorithm that identifies conformational epitopes based on the crystal structure of the protein. The structure used for the prediction consisted of chains I through N of PDB# 3CSY^2^. The original structure consists of 3 GP_1,2_ units (chains I through N) assembled as per their natural conformation, each bound to one F_ab_ unit (chains A through F) of the monoclonal antibody KZ52. The antibody KZ52, used to crystallize the glycoprotein, had previously been isolated from a human survivor of the 1995 Kikwit EBOV outbreak^25^. The original structure includes an additional GP_1,2_ subunit (chains O and P) bound to a single KZ52 F_ab_ fragment (chain G and H). Chains A through H, O, and P were removed using the PDB Editor software as they consist of KZ52 (A through H) and a GP_1,2_ monomer (O and P). The parameters for epitope prediction consisted of asking for epitopes on chains I and J, one of the three GP_1,2_ subunits, while keeping the other chains in the model so that residues on the surface of one monomer and in contact with another are properly considered to be buried.

### Affinity measurement

The BiAcore® experiments were carried out at room temperature using a BIAcore® 3000 machine with CM5 chips (GE Healthcare). For all the measurements, an HBS-EP buffer consisting of 10 mM HEPES, pH 7.5, 150 mM NaCl and 0.005% v/v Tween 20 was used, and all proteins were first exchanged to the same buffer by gel filtration. Each antibody was immobilized on the chip at approximately 300 response units (RU). Gradient concentrations of EBOV GP (0, 0.13, 0.26, 0.52, 1.04, 2.1, 4.2 and 8.4 μM) were then used to flow over the chip surface at 30 μl/min. After each cycle, the sensor surface was re-generated via a short treatment using 10 mM NaOH. The binding kinetics were recorded and analyzed with the software BIAevaluation® Version 4.1 using the 1:1 Langmuir binding model.

### Cross-inhibition assay

Cross-inhibition of 1H3, 2G4 and 4G7 was assessed by competitive ELISA. Signal was detected from human-mouse chimeric antibodies, produced as described previously. The mouse antibody 3H1 was used as a non-specific competitor, it is directed against the Marburg virus GP.

All ELISA steps were carried out in a volume of 30 μl. Costar EIA/RIA half-area microtiter plates were coated with sucrose-purified EBOV Kikwit at 5.8 μg of protein/well overnight at 4°C. The plates were blocked for 1 hr with 5% skim milk in PBS. The first antibody incubation was performed with mouse antibodies for 1 hr at a concentration of 0.2 μg/ml. The second antibody incubation was performed with the chimeric antibodies at a concentration of 0.2 μg/ml. The dilutions were chosen to produce an optical density at 405 nm (OD_405_) of approximately 1 with the chimeric antibodies alone. The secondary incubation was performed using a 1:2000 dilution of goat anti-human IgG (KPL) for 1 hr at 37°C. The plates were read after an incubation of 30 min with ABTS HRP substrate (KPL), at a wavelength of 405 nm on an VersaMax spectrophotometer. Three washes with PBS 0.1% Tween-20 were performed between each incubation step. Samples were run in triplicate and the average absorbance was reported. Inhibition was calculated as: 100 * (OD with blocking mAb/OD chimeric alone).

### Neutralization assays

The fluorescent neutralization assay was performed in 96-well tissue culture plates (Corning) seeded with Vero E6 cells, calculated to grow to 85–90% confluence on the day of infection. One hundred (100) PFU of VSVΔG-EBOVGP-eGFP or EBOV/May-eGFP were pre-incubated with the indicated concentrations of mAb (11 concentrations from 0.1 to 100 μg/ml) for 1 hr at 37°C in plain DMEM. The virus antibody mixture was then applied in triplicate to the VeroE6 cells per antibody concentration. Infection was carried out for 1 hr at 37°C, 5% CO_2_, removed and replaced with DMEM + 2% FBS. Plates were incubated for 24 (VSVΔG-EBOVGP-eGFP) or 48 hr (EBOV-eGFP), before fixation with 10% phosphate-buffered formalin (Fisher). Fluorescent plaques were counted using an iSpot FluoroSpot Reader System (Advanced Imaging Devices). The 50% EBOV neutralization concentration was calculated based on a one-phase decay curve fitted to the EBOV/May-eGFP neutralization curves using GraphPad Prism 5.

### Generation of escape mutants

In order to generate escape mutants, five 1/10 dilutions of VSVΔG-EBOVGP starting at 2.5 × 10^4^ PFU/ml VSVΔG-EBOVGP (final concentrations 2.5 × 10^4^, 2.5 × 10^3^, 2.5 × 10^2^, 2.5 × 10^1^, 2.5 × 10^0^ PFU/ml) were incubated at 37°C for 1 hr in the presence of 25, 12.5, or 10 μg/ml of 1H3, 2G4, or 4G7, respectively. Vero E6 cells in a 6-well plate were infected with the virus-antibody mixture for 1 hr, and then cultured in DMEM 2% FBS supplemented with 25, 12.5, or 10 μg/ml of the same antibody. VSVΔG-EBOVGP was allowed to grow for up to 11 days. When a dilution showed an 80% of cytopathic effect (CPE), it was harvested and frozen at −80°C. The highest viral dilution showing growth was used in the next step of the experiment. The supernatant harvested from the previous step was put through 2 additional rounds of selection using the same protocol, but in the presence of the same antibody at 50 μg/ml. The supernatant from the third round was used for RNA isolation using a QIAamp Viral RNA mini kit (Qiagen) and the region of the VSV genome containing the EBOV GP was amplified using a Qiagen One-Step RT-PCR kit using the primers listed in Supplementary Table 1. The PCR product was purified using a Qiagen PCR purification kit and sent for sequencing with the same primers. The GP sequences were assembled in SeqMan v9 and aligned using MEGAlign v9.

## Supplementary Material

Supplementary Information

## Figures and Tables

**Table 1:** Summary of the properties of the ZMAb antibodies

							% identity with germline sequence§	
mAb	Class	Neutralizing titer [μg/ml]*	Affinity [M]	J-gene‡	D-gene‡	Closest IgG germline gene‡	Nucleotide	Amino acid
V_H_								
1H3	IgG2a	27.6	2.11e-7	JH2	DSP2.4	VHSM7.a3.93	97.3	96.9
2G4	IgG2b	0.139	4.27e-7	JH4	DSP2.8	VHJ606.a.127#**	96.3	95.0
4G7	IgG2a	0.135	2.15e-7	JH3	DFL16.1	J558.12	95.9	92.9
V_L_								
1H3	κ	27.6	2.11e-7	JK2		at4	98.9	98.9
2G4	κ	0.139	4.27e-7	JK2		12-46	97.5	94.7
4G7	κ	0.135	2.15e-7	JK4		12-44	97.5	96.8

*Calculated as the 50% neutralization concentration based on a One-phase Decay curve fitted to the EBOV-eGFP neutralization data.

**The “#” sign is part of the name of the germline gene.

^‡^Germline genes were determined by using the IgBLAST tool.

^§^Based on nucleotide and amino acid alignments in MEGAlign v9.

**Table 2:** Cross-inhibition between the monoclonal antibodies

		Signal from		
		1H3	2G4	4G7
**Blocking mAb**	**1H3**	60	104	97
	**2G4**	96	64	66
	**4G7**	91	44	41
	**3H1**	114	102	106
