# Supplementary material for: Molecular Characterization of the Monoclonal Antibodies Composing ZMAb: A Protective Cocktail Against Ebola Virus
Source: Sci Rep. 2014 Nov 6;4:6881. doi: 10.1038/srep06881 (PMC5381473; doi:10.1038/srep06881)
Supplement: Supplementary Information [file srep06881-s1.docx]

**Molecular Characterization of the Monoclonal Antibodies Composing ZMAb: A Protective Cocktail Against Ebola Virus**

Jonathan Audet^1^, Gary Wong^2^, Han Wang^3^, Guangwen Lu^3^, George F Gao^3^, Gary Kobinger^1,2,4,5^, Xiangguo Qiu^2^*

| Primer name | Direction | Sequence | Sequencing pair |
| --- | --- | --- | --- |
| V135 | F | tgtttggcctgattgtcgag | 0 |
| 1F | F | CAGGTCCGGTGTCCCACCAAAGG | 1 |
| 3F | F | CGCTGAAGGTGTCGTTGCA | 2 |
| 4R | R | TGCAACGACACCTTCAGCG | 0 |
| 6R | R | GTGAATCTTGATTCAAGTTTTACGTA | 1 |
| 5F | F | CTGCTCCAGCTGAATGAGAC | 3 |
| 7F | F | GTTCAAGTGCACAGTCAAGG | 4 |
| 8R | R | CCTTGACTGTGCACTTGAAC | 2 |
| 9F | F | GAGCAAGGGTACCGACCTCC | 5 |
| 10R | R | GGAGGTCGGTACCCTTGCTC | 3 |
| 11F | F | GGATGAAGGTGCTGCAATCG | 6 |
| 12R | R | CGATTGCAGCACCTTCATCC | 4 |
| 14R | R | GTTCGATACAGCAGTCCG | 5 |
| S110 | R | gtaatcagcatgattcaagtacg | 6 |

Supplementary Table 1: Primers used to sequence VSVΔG-EBOV escape mutants
